# Polynomial-Time Algorithm for Learning Optimal BFS-Consistent Dynamic Bayesian Networks

**DOI:** 10.3390/e20040274

**Published:** 2018-04-12

**Authors:** Margarida Sousa, Alexandra M. Carvalho

**Affiliations:** Instituto de Telecomunicações, Instituto Superior Técnico, Universidade de Lisboa, 1049-001 Lisboa, Portugal

**Keywords:** dynamic Bayesian networks, optimum branching, score-based learning, theoretical-information scores

## Abstract

Dynamic Bayesian networks (DBN) are powerful probabilistic representations that model stochastic processes. They consist of a prior network, representing the distribution over the initial variables, and a set of transition networks, representing the transition distribution between variables over time. It was shown that learning complex transition networks, considering both intra- and inter-slice connections, is NP-hard. Therefore, the community has searched for the largest subclass of DBNs for which there is an efficient learning algorithm. We introduce a new polynomial-time algorithm for learning optimal DBNs consistent with a breadth-first search (BFS) order, named bcDBN. The proposed algorithm considers the set of networks such that each transition network has a bounded in-degree, allowing for *p* edges from past time slices (inter-slice connections) and *k* edges from the current time slice (intra-slice connections) consistent with the BFS order induced by the optimal tree-augmented network (tDBN). This approach increases exponentially, in the number of variables, the search space of the state-of-the-art tDBN algorithm. Concerning worst-case time complexity, given a Markov lag *m*, a set of *n* random variables ranging over *r* values, and a set of observations of *N* individuals over *T* time steps, the bcDBN algorithm is linear in *N*, *T* and *m*; polynomial in *n* and *r*; and exponential in *p* and *k*. We assess the bcDBN algorithm on simulated data against tDBN, revealing that it performs well throughout different experiments.

## 1. Introduction

Bayesian networks (BN) represent, in an efficient and accurate way, the joint probability of a set of random variables [1]. Dynamic Bayesian networks (DBN) are the dynamic counterpart of BNs and model stochastic processes [2]. DBNs consist of a prior network, representing the distribution over the initial attributes, and a set of transition networks, representing the transition distribution between attributes over time. They are used in a large variety of applications such as protein sequencing [3], speech recognition [4] and clinical forecasting [5].

The problem of learning a BN given data consists in finding the network that best fits the data. In a score-based approach, a scoring criterion is considered, which measured how well the network fits the data [6,7,8,9,10]. In this case, learning a BN reduces to the problem of finding the network that maximizes the score, given the data. Methods for learning DBNs are simple extensions of those considered for BNs [2]. Not taking into account the acyclicity constraints, it was proved that learning BNs does not have to be NP-hard [11]. This result can be applied to DBNs, not considering the intra-slice connections, as the resulting unrolled graph, which contains a copy of each attribute in each time slice, is acyclic. Profiting from this result, a polynomial-time algorithm for learning optimal DBN was proposed using the Mutual Information Tests (MIT) [12]. However, none of these algorithms learns general *m*th-order Markov DBNs such that each transition network has inter- and intra-slice connections. More recently, a polynomial-time algorithm was proposed that learns both the inter- and intra-slice connections in a transition network [13]. The search space considered, however, is restricted to the tree-augmented network structures, resulting in the so-called tDBN.

By looking into lower-bound complexity results for learning BNs, it is known that learning tree-like structures is polynomial [14]. However, learning 2-polytrees is already NP-hard [15]. Learning efficiently structures richer than branchings (a.k.a. tree-like structures) has eluded the community, that resorted to use heuristic approaches. Carvalho et al. [16] suggested to search over graphs consistent with the topological order of an optimal branching. The advantage of this approach is that the search space increased exponentially with respect to branchings, while keeping the learning complexity in polynomial time. Later, the breadth-first search (BFS) order of an optimal branching was also considered [17], further improving the previous results in terms of search space.

In this paper, we propose a generalization of the tDBN algorithm, considering DBNs such that each transition network is consistent with the order induced by the BFS order of the optimal branching of the tDBN network, that we call bcDBN. Furthermore, we prove that the search space increases exponentially, in the number of attributes, comparing with the tDBN algorithm, while running in polynomial time.

We start by reviewing the basic concepts of Bayesian networks, dynamic Bayesian networks and their learning algorithms. Then, we present the proposed algorithm and the experimental results. The paper concludes with a brief discussion and directions for future work.

## 2. Bayesian Networks

Let *X* denote a discrete random variable that takes values over a finite set X. Furthermore, let $X=\;(X_{1},\ldots ,X_{n})$ represent an *n*-dimensional random vector, where each $X_{i}$ takes values in $X_{i}=\;\left\{x_{i1},\ldots ,x_{ir_{i}}\right\}$, and P(x) denotes the probability that X takes the value x. A Bayesian network encodes the joint probability distribution of a set of *n* random variables $\left\{X_{1},\ldots ,X_{n}\right\}$ [1].

**Definition** **1** (Bayesian Network). *A n-dimensional Bayesian Network (BN) is a triple B=(X,G,Θ), where:*

- $X=(X_{1},\ldots ,X_{n})$ and each random variable $X_{i}$ takes values in the set $\left\{x_{i1},\ldots ,x_{ir_{i}}\right\}$, where $x_{ik}$ denotes the k-th value $X_{i}$ takes.
- *G=(X,E) is a directed acyclic graph (DAG) with nodes in X and edges E representing direct dependencies between the nodes.*
- *$\Theta ={\left\{\Theta_{ijk}\right\}}_{i\in 1\ldots n,j\in 1...q_{i},k\in 1\ldots ..,r_{i}}$ encodes the parameters of the network G, a.k.a. conditional probability tables (CPT):*
  (1) $$\Theta_{ijk}=P_{B}\left(X_{i}=x_{ik}|\Pi_{X_{i}}=w_{ij}\right),$$
  *where $\Pi_{X_{i}}$ denotes the set of parents of $X_{i}$ in the network G and $w_{ij}$ is the j-th configuration of $\Pi_{X_{i}}$, among all possible configurations given by $\left\{w_{i1},\ldots ,w_{iq_{i}}\right\}$, with $q_{i}=\prod_{X_{j}\in \Pi_{X_{i}}}r_{j}$ denoting the total number of parent configurations.*

A BN *B* induces a unique joint probability distribution over X given by:

(2) $$P_{B}\left(X_{1},\ldots ,X_{n}\right)=\prod_{i=1}^{n}P_{B}\left(X_{i}|\Pi_{X_{i}}\right).$$

Let $N_{ijk}$ be the number of instances in data set *D* of size *N*, where variable $X_{i}$ takes the value $x_{ik}$ and the set of parents $\Pi_{X_{i}}$ takes the configuration $w_{ij}$. Denote the number of instances in *D* where the set of parents $\Pi_{X_{i}}$ takes the configuration $w_{ij}$ by

$$N_{ij}=\sum_{k=1}^{r_{i}}N_{ijk}.$$

Observe that,

$$X_{i}|\Pi_{X_{i}}∼\mathrm{Multinomial}\left(N_{ij},\theta_{ij1},\ldots ,\theta_{ijr_{i}}\right),$$

for i∈{1,…,n} and $j\in \{1,\ldots ,q_{i}\}$.

Intuitively, the graph of a BN can be viewed as a network structure that provides the skeleton for representing the joint probability compactly in a factorized way, and making inferences in the probabilistic graphical model provides the mechanism for gluing all these components back together in a probabilistic coherent manner [18].

**Figure 1:**
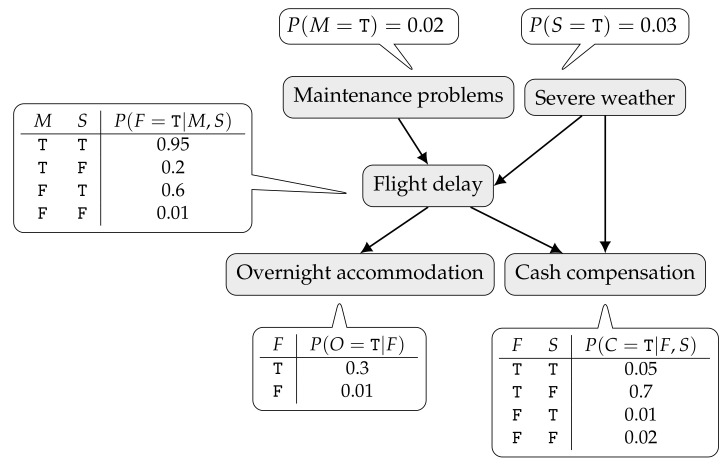
A BN example regarding airline regulations with conditional probability tables.

An example of a BN is depicted in Figure 1. It describes cash compensation and overnight accommodation to air passengers in the event of long flight delays. A flight may be delayed due to aircraft maintenance problems or severe weather (hurricane, blizzard, etc.). Whenever the delay is not caused by an external event to the airline company, a passenger may be entitled to a monetary compensation. Regardless of the cause, if the delay is long enough, the passenger might be offered an overnight accommodation. As a result of the dependences encoded by the graph, the joint probability distribution of the network can be factored as

P(M,S,F,O,C)=P(M)P(S)P(F|M,S)P(O|F)P(C|F,S),

where only the first letter of a variable name is used: *M*—Maintenance problems; *S*—Severe weather; *F*—Flight delay; *O*—Overnight accommodation; and *C*—Cash compensation. In this simple example, all variables are Bernoulli (ranging over T and F). Inside the callouts only the CPTs for variables taking the value T are given.

## 3. Learning Bayesian Networks

Learning a Bayesian network is two-fold: parameter learning and structure learning. When learning the parameters, we assume the underlying graph *G* is given, and our goal is to estimate the set of parameters of the network Θ. When learning the structure, the goal is to find a structure *G*, given only the training data. We assume data is complete, i.e., each instance is fully observed, there are no missing values nor hidden variables, and the training set $D=\{x_{1},\ldots ,x_{N}\}$ is given by a set of *N* i.i.d. instances. Using general results of the maximum likelihood estimate in a multinomial distribution we get the following estimate for the parameters of a BN *B*:

(3) $${\hat{\theta }}_{ijk}=\frac{N_{ijk}}{N_{ij}},$$

that is denoted by observed frequency estimate (OFE).

In score-based learning, a scoring function ϕ:S×X→R is required to measure how well a BN *B* fits the data *D* (where S denotes the search space). In this case, the learning procedure can be extremely efficient if the employed score is decomposable. A scoring function ϕ is said to be decomposable if the score can be expressed as a sum of local scores that depends only on each node and its parents, that is, in the form:

$$\phi \left(B,D\right)=\sum_{i=1}^{n}\phi_{i}\left(\Pi_{X_{i}},D\right).$$

Well-known decomposable scores are divided in two classes: Bayesian and information-theoretical. Herein, we focus only on two information-theoretical criteria, namely Log-Likelihood (LL) and Minimum Description Length (MDL) [19]. Information-theoretical scores are based on the compression achieved to describe the data, given an optimal code induced by a probability distribution encoded by a BN.

The LL is given by:

(4) $$LL\left(B|D\right)=\sum_{i=1}^{n}\sum_{j=1}^{q_{i}}\sum_{k=1}^{r_{i}}N_{ijk}\log\left(\theta_{ijk}\right).$$

This criterion favours complete network structures, and does not generalize well, leading to the overfitting of the model to the training data. The MDL criterion, proposed by Rissanen [19], imposes that the parameters of the model, ignored in the LL score, must also be accounted. The MDL score for learning BNs is defined by:

(5) $$MDL\left(B|D\right)=LL\left(B|D\right)-\frac{1}{2}\ln\left(N\right)\left|B\right|,$$

where |B| corresponds to the number of parameters Θ of the network, given by:

(6) $$\left|B\right|=\sum_{i=1}^{n}\left(r_{i}-1\right)q_{i}.$$

The penalty introduced by MDL creates a trade off between fitness and model complexity, providing a model selection criterion robust to overfitting.

The structure learning reduces to an optimization problem: given a scoring function, a data set, a search space and a search procedure, find the network that maximizes this score. Denote the set of BNs with *n* random variables by $B_{n}$.

**Definition** **2** (Learning a Bayesian Network). Given a data $D=\{x_{1},\ldots ,x_{N}\}$ and a scoring function ϕ, the problem of learning a Bayesian network is to find a Bayesian network $B\in B_{n}$ that maximizes the value ϕ(B,D).

The space of all Bayesian networks with *n* nodes has a superexponential number of structures, $2^{O(n^{2})}$. Learning general Bayesian networks is a NP-hard problem [20,21,22]. However, if we restrict the search space S to tree-like structures [14,23] or to networks with bounded in-degree and a known ordering over the variables [24], it is possible to obtain a global optimal solution for this problem. Polynomial-time algorithms to learn BNs with underlying consistent *k*-graphs (C*k*G) [16] and breadth-first search consistent *k*-graphs (BC*k*G) [17] network structures were proposed. The sets of C*k*G and BC*k*G graphs are exponentially larger, in the number of variables, when compared with branchings [16,17].

**Definition** **3** (*k*-graph). *A k-graph is a graph where each node has in-degree at most k.*

**Definition** **4** (Consistent *k*-graph). Given a branching R over a set of nodes V, a graph G=(V,E) is said to be a consistent k-graph (CkG) w.r.t. R if it is a k-graph and for any edge in E from $X_{i}$ to $X_{j}$ the node $X_{i}$ is in the path from the root of R to $X_{j}$.

**Definition** **5** (BFS-consistent *k*-graph). Given a branching R over a set of nodes V, a graph G=(V,E) is said to be a BFS-consistent k-graph (BCkG) w.r.t. R if it is a k-graph and for any edge in E from $X_{i}$ to $X_{j}$ the node $X_{i}$ is visited in breadth-first search (BFS) of R before $X_{j}$.

**Figure 2:**
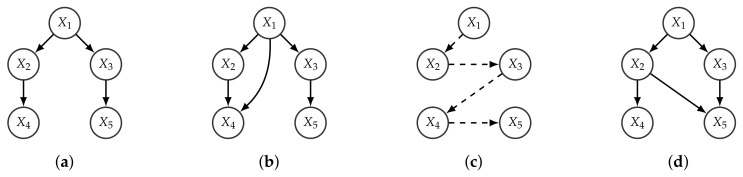
Given the branching *R* represented in (**a**); (**b**) represents a consistent 2-graph with respect to *R*; (**c**) represents the BFS of R and (**d**) represents a BFS-consistent 2-graph of *R* (not consistent with *R*).

Observe that the order induced by the optimal branching might be partial, while its BFS order is always total (and refines it). Given a BFS-consistent *k*-graph, there can only exist an edge from $X_{i}$ to $X_{j}$ if $X_{i}$ is less than or as deep as $X_{j}$ in *R*. We assume that if i<j and $X_{i}$ and $X_{j}$ are at the same level, then the BFS over *R* reaches $X_{i}$ before $X_{j}$. An example is given in Figure 2.

## 4. Dynamic Bayesian Networks

Dynamic Bayesian networks (DBN) model the stochastic evolution of a set of random variables over time [2]. Consider the discretization of time in time slices given by the set T={0,…,T}. Let $X\left[t\right]=(X_{1}\left[t\right],\ldots ,X_{n}\left[t\right])$ be a random vector that denotes the value of the set of attributes at time *t*. Furthermore, let $X[t_{1}:t_{2}]$ denote the set of random variables X for the interval $t_{1}\leq t\leq t_{2}$. Consider a set of individuals H measured over *T* sequential instants of time. The set of observations is represented as ${\left\{x^{h}\left[t\right]\right\}}_{h\in H,t\in T}$, where $x^{h}\left[t\right]=\left(x_{1}^{h},\ldots ,x_{n}^{h}\right)$ is a single observation of *n* attributes, measured at time *t* and referring to individual *h*.

In the setting of DBNs the goal is to define a probability joint distribution over all possible trajectories, i.e., possible values for each attribute $X_{i}$ and instant *t*, $X_{i}\left[t\right]$. Let $P(X\left[t_{1}:t_{2}\right])$ denote the joint probability distribution over the trajectory of the process from $X[t_{1}]$ to $X[t_{2}]$. The space of possible trajectories is very large, therefore in order to define a tractable problem it is necessary to make assumptions and simplifications.

Observations are viewed as i.i.d. samples of a sequence of probability distributions ${\left\{P_{\theta [t]}\right\}}_{t\in T}$. For all individuals h∈H, and a fixed time *t*, the probability distribution is considered constant, i.e., $x^{h}\left[t\right]∼P_{\theta [t]},h\in H$. Using the chain rule the joint probability over X is given by:

$$P\left(X[0:T]\right)=P\left(X[0]\right)\prod_{t=0}^{T-1}P\left(X[t+1]|X[0:t]\right).$$

**Definition** **6** (*m*th-Order Markov assumption). *A stochastic process over X satisfies the mth-order Markov assumption if, for all t≥0*

(7) $$P\left(X[t+1]|X[0:t]\right)=P\left(X[t+1]|X[t-m+1:t]\right).$$

*In this case m is called the Markov lag of the process.*

If all conditional probabilities in Equation (7) are invariant to shifts in time, that is, are the same for all t∈T, then the stochastic process is called a stationary *m*th-order Markov process.

**Definition** **7** (First-order Markov DBN). *A non-stationary first-order Markov DBN consists of:*

- A prior network $B^{0}$, which specifies a distribution over the initial states X[0].
- A set of transition networks $B_{t}^{t+1}$ over the variables X[t:t+1], representing the state transition probabilities, for 0≤t≤T−1.

We denote by $G_{t+1}$ the subgraph of $B_{t}^{t+1}$ with nodes X[t+1], that contains only the intra-slice dependencies. The transition network $B_{t}^{t+1}$ has the additional constraint that edges between slices (inter-slice connections) must flow forward in time. Observe that in the case of a first-order DBN a transition network encodes the inter-slice dependencies (from time transitions t→t+1) and intra-slice dependencies (in the time slice t+1).

**Figure 3:**
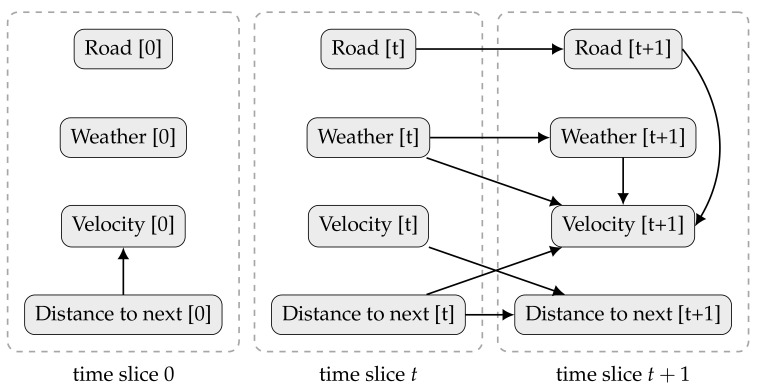
A simple example of a first-order Markov stationary DBN. On the left, the prior network $B_{0}$, for t=0. On the right, a two-slice transition network $B_{t}^{t+1}$.

**Figure 4:**
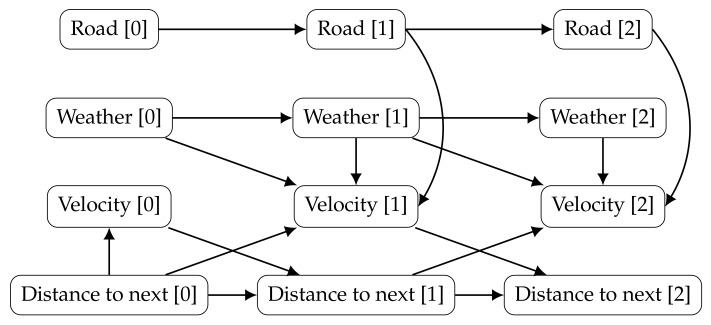
The DBN example from Figure 3 is unrolled for the first three time slices.

Figure 3 shows an example of a DBN, aiming to infer a driver behaviour. The model describes the state of a car, including its velocity and distance to the following vehicle, as well as, the weather and the type of road (highway, arterial, local road, etc.). In the beginning, the speed depends only if there is a car nearby. After that, the velocity depends on: (i) the previous weather (the road might be icy because it snowed last night); (ii) the current weather (it might be raining now); (iii) how close the car was from another (if it gets too close the driver might need to break); and (iv) the current type of road (with different velocity limits). The current distance to the following car depends on the previous car velocity and on the previous distance to the next vehicle. Figure 4 joins the prior and transition networks and extends the unrolled DBN to a third time slice.

Learning DBNs, considering no hidden variables or missing values, i.e., considering a fully observable process, reduces simply to applying the methods described for BNs for each transition of time [25]. Several algorithms for learning DBNs are concerned with identifying inter-slice connections only, disregarding intra-slice dependencies or assuming they are given by some prior network and kept fixed over time [11,12,26]. Recently, a polynomial-time algorithm was proposed that learns both the inter and intra-slice connections in a transition network [13]. However, the search space is restricted to tree-augmented network structures (tDBN), i.e., acyclic networks such that each attribute has one parent from the same time slice, but can have at most *p* parents from the previous time slices.

**Definition** **8** (Tree-augmented DBN). A dynamic Bayesian network is called tree-augmented (tDBN) if for each transition network {t−m+1,…,t}→t+1 each attribute $X_{i}\left[t+1\right]$ has exactly one parent in the time slice t+1, except the root, and at most p parents from the preceding time slices {t−m+1,…,t}.

## 5. Learning Consistent Dynamic Bayesian Networks

We introduce a polynomial-time algorithm for learning DBNs such that: the intra-slice network has in-degree at most *k* and is consistent with the BFS order of the tDBN; the inter-slice network has in-degree of at most *p*. The main idea of this approach is to add dependencies that were lost due to the tree-augmented restriction of the tDBN and, furthermore, remove irrelevant ones that might be present because a connected graph was imposed. Moreover, we also consider the BFS order of the intra-slice network as an heuristic for a causality order between variables. We make this concept rigorous with the following definition.

**Definition** **9** (BFS-consistent *k*-graph DBN). *A dynamic Bayesian network is called BFS-consistent k-graph (bcDBN) if for each intra-slice network $G_{t+1}$, with t∈{0,…,T−1}, the following holds:*

- *$G_{t+1}$ is a k-graph, i.e., each node has in-degree at most k;*
- *Given an optimal branching $R_{t+1}$ over the set of nodes X[t+1], for every edge in $G_{t+1}$ from $X_{i}\left[t+1\right]$ to $X_{j}\left[t+1\right]$, the node $X_{i}\left[t+1\right]$ is visited in the BFS of $R_{t+1}$ before $X_{j}\left[t+1\right]$.*

*Moreover, each node $X_{i}\left[t+1\right]$ has at most p parents from previous time slices.*

Before we present the learning algorithm, we need to introduce some notation, namely, the concept of ancestors of a node.

**Definition** **10** (Ancestors of a node). *The ancestors of a node $X_{i}$ in time slice t+1, denoted by $\alpha_{i,t+1}^{BFS}$ , are the set of nodes in slice t+1 connecting the root of the BFS of an optimal branching $R_{t+1}$ and $X_{i}\left[t+1\right]$.*

We will now describe briefly the proposed algorithm for learning a transition network of a *m*th-order bcDBN. Let $P_{\leq p}\left(X\left[t-m+1:t\right]\right)$ be the set of subsets of X[t−m+1:t] of cardinality less than or equal to *p*. For each node $X_{i}\left[t+1\right]\in X\left[t+1\right]$, the optimal set of past parents ($X_{ps}\in P_{\leq p}\left(X\left[t-m+1:t\right]\right)$) and maximum score ($s_{i}$) is found,

(8) $$s_{i}=\max_{X_{ps}\left[t-m+1:t\right]\in P_{\leq p}\left(X\left[t-m+1:t\right]\right)}\phi_{i}\left(X_{ps}\left[t-m+1:t\right],D_{t-m+1}^{t+1}\right),$$

where $\phi_{i}$ is the local contribution of $X_{i}\left[t+1\right]$ for the overall score ϕ and $D_{t-m+1}^{t+1}$ is the subset of observations concerning the time transition t−m+1→t+1. For each possible edge in t+1, $X_{j}\left[t+1\right]\to X_{i}\left[t+1\right]$, the optimal set of past parents and maximum score ($s_{ij}$) is determined,    

(9) $$s_{ij}=\max_{X_{ps}\left[t-m+1:t\right]\in P_{\leq p}\left(X\left[t-m+1:t\right]\right)}\phi_{i}\left(X_{ps}\left[t-m+1:t\right]\cup \left\{X_{j}\left[t+1\right]\right\},D_{t-m+1}^{t+1}\right).$$

We note that the set $X_{ps}\left[t-m+1:t\right]$ that maximizes Equations (8) and (9) needs not to be the same. The one in Equation (8) refers to the best set of *p* parents from past time slices, and the one in Equation (9) concerns the best set of *p* parents from the past time slices when $X_{j}\left[t+1\right]$ is also a parent of $X_{i}\left[t+1\right]$.

A complete directed graph is built such that each edge $X_{j}\left[t+1\right]\to X_{i}\left[t+1\right]$ has the following weight,

(10) $$e_{ij}=s_{ij}-s_{i},$$

that is, the gain in the network score of adding $X_{j}\left[t+1\right]$ as a parent of $X_{i}\left[t+1\right]$. Generally $e_{ij}\neq e_{ji}$, as the edge $X_{i}\left[t+1\right]\to X_{j}\left[t+1\right]$ may account for the contribution from the inter-slice parents and, in general, inter-slice parents of $X_{i}\left[t+1\right]$ and $X_{j}\left[t+1\right]$ are not the same. Therefore, Edmond’s algorithm is applied to obtain a maximum branching for the intra-slice network [27]. In order to obtain a total order, the BFS order of the output maximum branching is determined and the set of candidate ancestors $\alpha_{i,t+1}^{BFS}$ is computed. For node $X_{i}\left[t+1\right]$, the optimal set of past parents $X_{ps}\left[t-m+1:t\right]$ and intra-slice parents, denoted by $X_{ps}\left[t+1\right]$, are obtained in a one-step procedure by finding

(11) $$\max_{X_{ps}\left[t-m+1:t\right]\in P_{\leq p}\left(X\left[t-m+1:t\right]\right)}\max_{X_{ps}\left[t+1\right]\in P_{\leq k}\left(\alpha_{i,t+1}^{BFS}\right)}\phi_{i}\left(X_{ps}\left[t-m+1:t\right]\cup X_{ps}\left[t+1\right],D_{t-m+1}^{t+1}\right),$$

where $P_{\leq k}\left(\alpha_{i,t+1}^{BFS}\right)$ is the set of all subsets of $\alpha_{i,t+1}^{BFS}$ of cardinality less than or equal to *k*. Note that, if $X_{i}\left[t+1\right]$ is the root, $P_{\leq k}\left(\alpha_{i,t+1}^{BFS}\right)=\left\{\emptyset \right\}$, so the set of intra-slice parents $X_{ps}\left[t+1\right]$ of $X_{i}\left[t+1\right]$ is always empty.

The pseudo-code of the proposed algorithm is given in Algorithm 1. As parameters, the algorithm needs: a dataset *D*, a Markov lag *m*, a decomposable scoring function ϕ, a maximum number of inter-slice parents *p* and a maximum number of intra-slice parents *k*.

**Algorithm 1** Learning optimal *m*th-order Markov bcDBN
1:**for** each transition {t−m+1,…,t}→t+1 **do**2: Build a complete directed graph in X[t+1].3: Weight all edges $X_{j}\left[t+1\right]\to X_{i}\left[t+1\right]$ of the graph with $e_{ij}$ as in Equation (10) (Algorithm 2).4: Apply Edmond’s algorithm to the intra-slice network, to obtain an optimal branching.5: Build the BFS order of the output optimal branching.6: **for** all nodes $X_{i}\left[t+1\right]$ **do**7:  Compute the set of parents of $X_{i}\left[t+1\right]$ as in Equation (11) (Algorithm 3).8: **end for**9:**end for**10:Collect the transition networks to obtain the optimal bcDBN structure.


The algorithm starts by building the complete directed graph in Step 2, after which the graph is weighted according to Equation (10); this procedure is described in detail in Algorithm 2. The Edmonds’ algorithm is then applied to the intra-slice network, resulting from that an optimal branching (Step 4). The BFS order of this branching is computed (Step 5) and the final transition network is redefined to be consistent with it. This is done by computing the parents of $X_{i}\left[t+1\right]$ given by Equation (11) (Steps 6–7), further detailed in Algorithm 3.

**Theorem** **1.** *Algorithm 1 finds an optimal mth-order Markov bcDBN, given a decomposable scoring function ϕ, a set of n random variables, a maximum intra-slice network in-degree of k and a maximum inter-slice network in-degree of p.*

**Proof.**  Let *B* be the optimal bcDBN and $B^{\prime }$ be the DBN output of Algorithm 1. Consider without loss of generality the time transition {t−m+1,…,t}→t+1. The proof follows by contradiction, assuming that the score of $B^{\prime }$ is lower than *B*. The contradiction found is the following: the optimal branching algorithm applied to the intra-slice graph, Step 4 of Algorithm 1, outputs an optimal branching; moreover, all sets of parents with cardinality of at most *k* consistent with the BFS order of the optimal branching and all sets of parents from the previous time slices with cardinality of at most *p* are checked in the for-loop at Step 6. Therefore, the optimal set of parents is found for each node. Finally, note that the selected graph is acyclic since: (i) in the intra-slice network the graph is consistent with a total order (so no cycle can occur); and (ii) in the inter-slice network there are only dependencies from previous time slices to the present one (and not on the other way). ☐

**Algorithm 2** Compute all the weights $e_{ij}$
1:**for** all nodes $X_{i}\left[t+1\right]$ **do**2: Let $s_{i}=-\infty$.3: **for** $X_{ps}\left[t-m+1:t\right]\in P_{\leq p}\left(X\left[t-m+1:t\right]\right)$ **do**
4:  **if** $\phi_{i}\left(X_{ps}\left[t-m+1:t\right],D_{t-m+1}^{t+1}\right)>s_{i}$ **then**
5:   Let $s_{i}=\phi_{i}\left(X_{ps}\left[t-m+1:t\right],D_{t-m+1}^{t+1}\right)$.6:  **end if**
7: **end for**
8: **for** all nodes $X_{j}\left[t+1\right]\neq X_{i}\left[t+1\right]$ **do**9:  Let $s_{ij}=-\infty$.10:  **for** $X_{ps}\left[t-m+1:t\right]\in P_{\leq p}\left(X\left[t-m+1:t\right]\right)$ **do**
11:   **if** $\phi_{i}\left(X_{ps}\left[t-m+1:t\right]\cup \left\{X_{j}\left[t+1\right]\right\},D_{t-m+1}^{t+1}\right)>s_{ij}$ **then**
12:    Let $s_{ij}=\phi_{i}\left(X_{ps}\left[t-m+1:t\right]\cup \left\{X_{j}\left[t+1\right]\right\},D_{t-m+1}^{t+1}\right)$.13:   **end if**
14:  **end for**
15: **end for**
16: Let $e_{ij}=s_{ij}-s_{i}$.17:**end for**


**Algorithm 3** Compute the set of parents of $X_{i}\left[t+1\right]$
1:Let max=−∞.2:**for** $X_{ps}\left[t-m+1:t\right]\in P_{\leq p}\left(X\left[t-m+1:t\right]\right)$ **do**
3: **for** $X_{ps}\left[t+1\right]\in P_{\leq k}\left(\alpha_{i,t+1}^{BFS}\right)$ **do**
4:  **if** $\phi_{i}\left(X_{ps}\left[t-m+1:t\right]\cup X_{ps}\left[t+1\right],D_{t-m+1}^{t+1}\right)>\max$ **then**
5:   Let max = $\phi_{i}\left(X_{ps}\left[t-m+1:t\right]\cup X_{ps}\left[t+1\right],D_{t-m+1}^{t+1}\right)$.6:   Let the parents of $X_{i}\left[t+1\right]$ be $X_{ps}\left[t-m+1:t\right]\cup X_{ps}\left[t+1\right]$.7:  **end if**
8: **end for**
9:**end for**

**Theorem** **2.** *Algorithm 1 takes time*

$$\max\{O\left(n^{p+3}{\left(m+1\right)}^{3}m^{p}r^{p+2}N\left(T-m+1\right)\right),O\left(n^{p+k+2}m^{p}\left(m+1\right)r^{p+k+1}N\left(T-m+1\right)\right)\},$$

*given a decomposable scoring function ϕ, a Markov lag m, a set of n random variables, a bounded in-degree of each intra-slice transition network of k, a bounded in-degree of each inter-slice transition network of p and a set of observations of N individuals over T time steps.*

**Proof.**  For each time transition {t−m+1,…,t}→t+1, in order to compute all weights $e_{ij}$ (Algorithm 2), it is necessary to iterate over all the edges, that takes time $O({\left(n\left(m+1\right)\right)}^{2})$. The number of subsets of parents from the preceding time slices with at most *p* elements is given by:

(12) |P≤p(X[t])|=∑i=0pnmi<∑i=0p(nm)i∈O((nm)p).

Calculating the score of each parent set (Step 11 of Algorithm 2), considering that the maximum number of states a variable may take is *r*, and that each variable has at most p+1 parents (*p* from the past and 1 in t+1), the number of possible configurations is given by $r^{p+2}$. The score of each configuration is computed over the set of observations $D_{t-m+1}^{t+1}$, therefore taking $O(\left(m+1\right)r^{p+2}nN)$. Applying Edmond’s optimal branching algorithm to the intra-slice network and computing its BFS order, in Steps 4 and 5, takes $O(n^{2})$ time. Hence, Steps 1–5 take time $O(n^{p+3}{\left(m+1\right)}^{3}m^{p}r^{p+2}N)$. Step 6 iterates over all nodes in time slice t+1, therefore iterates O(n) times. In Algorithm 3, Step 7, the number of subsets with at most *p* elements from the past and *k* elements from the present is upper bounded by $O({\left(nm\right)}^{p}n^{k})$. Computing the score of each configuration takes time complexity of $O(\left(m+1\right)nr^{p+k+1}N)$. Therefore Steps 6–9 take time complexity of $O(n^{p+k+2}m^{p}\left(m+1\right)r^{p+k+1}N)$. Algorithm 1 ranges over all T−m+1 time transitions, hence, takes time $\max\{O\left(n^{p+3}{\left(m+1\right)}^{3}m^{p}r^{p+2}N\left(T-m+1\right)\right)$, $O(\left(n^{p+k+2}m^{p}\left(m+1\right)r^{p+k+1}N\left(T-m+1\right)\right)\}$. ☐

**Theorem** **3.** *There are at least $2^{\left(nk-\frac{k^{2}}{2}-\frac{k}{2}-1\right)\left(T-m+1\right)}$ non-tDBN transition networks in the set of bcDBN structures, where n is the number of variables, T is the number of time steps considered, m is the Markov lag and k is the maximum intra-slice in-degree considered.*

**Proof.**  Consider without loss of generality the time transition {t−m+1,…,t}→t+1 and the optimal branching in t+1, $R_{t+1}$. Let $\left(V,\subseteq_{BFS}\right)$ be the total order induced by the BFS over $R_{t+1}$. For any two nodes $X_{i}\left[t+1\right]$ and $X_{j}\left[t+1\right]$, with i≠j, we say that node $X_{i}\left[t+1\right]$ is lower than $X_{j}\left[t+1\right]$ if $X_{i}\left[t+1\right]\subseteq_{BFS}X_{j}\left[t+1\right]$. The *i*-th node of $R_{t+1}$ has precisely i−1 lower nodes. When i>k, there are at least $2^{k}$ subsets of *V* with at most *k* lower nodes. When i≤k, only $2^{i-1}$ subsets of *V* with at most *k* lower nodes exist. Therefore, there are at least

$$\left(\prod_{i=k+1}^{n}2^{k}\right)\times \left(\prod_{i=1}^{k}2^{i-1}\right)=2^{nk-\frac{k^{2}}{2}-\frac{k}{2}}$$

BFS-consistent *k*-graphs.

Let $X_{R}$ be the root of $R_{t+1}$ and $X_{j}$ its child node. Let ∅ denote the empty set. $X_{R}$ and ∅ are the only possible ancestors of $X_{j}$. If ∅ is the optimal one, then the resultant graph will not be a tree-augmented network. Therefore there are at least

$$2^{nk-\frac{k^{2}}{2}-\frac{k}{2}-1}$$

non-tree-augmented graphs in the set of BFS-consistent *k*-graphs.

There are T−m+1 transition networks, hence, there are at least $2^{\left(nk-\frac{k^{2}}{2}-\frac{k}{2}-1\right)\left(T-m+1\right)}$ non-tDBN network structures in the set of bcDBN network structures. ☐

## 6. Experimental Results

We assess the merits of the proposed algorithm comparing it with one state-of-the-art DBN learning algorithm, tDBN [13]. Our algorithm was implemented in Java using an object-oriented paradigm and was released under a free software license (https://margaridanarsousa.github.io/learn_cDBN/). The experiments were run on an Intel^®^ Core™ i5-3320M CPU @ 2.60GHz×4 machine.

We analyze the performance of the proposed algorithm for synthetic data generated from stationary first-order Markov bcDBNs. Five bcDBN structures were determined, parameters were generated arbitrarily, and observations were sampled from the networks, for a given number of observations *N*. The parameters *p* and *k* were taken to be the maximum in-degree of the inter and intra-slice network, respectively, of the transition network considered.

**Figure 5:**
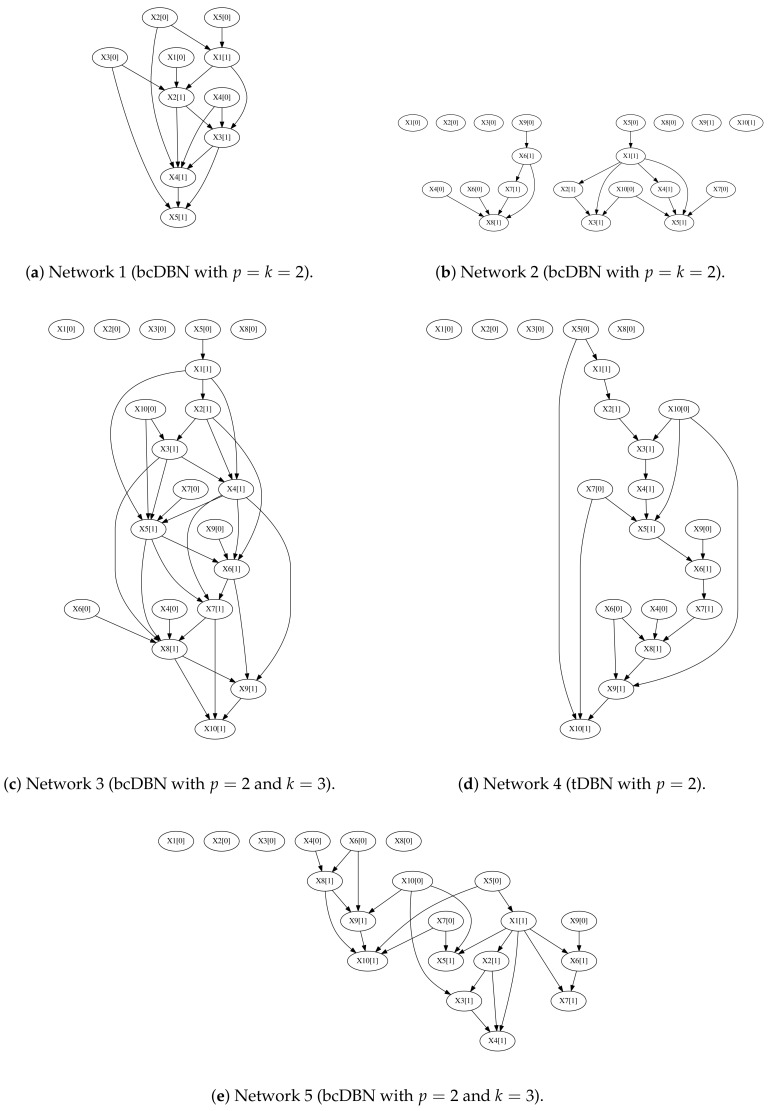
First-order Markov stationary transition networks considered in the experiments.

In detail, the five first-order Markov stationary transition networks considered were:

- one intra-slice complete bcDBN network with k=2 and at most p=2 parents from the previous time slice (Figure 5a);
- one incomplete bcDBN network, such that each node in t+1 has a random number of inter-slice (p=2) and intra-slice (k=2) parents between 0 and p+k≤4 (Figure 5b);
- two incomplete intra-slice bcDBN network (k=3) such that each node has at most p=2 parents from the previous time slice (Figure 5c,e);
- one tDBN (k=1), such that each node has at most p=2 parents from the previous time slice (Figure 5d).

The tDBN and bcDBN algorithms were applied to the resultant data sets, and the ability to learn and recover the original network structure was measured. We compared the original and recovered networks using the precision, recall and $F_{1}$ metrics:

$$\mathrm{precision}=\frac{TP}{TP+FP},\mathrm{recall}=\frac{TP}{TP+FN}\;\mathrm{and}\;F_{1}=2\times \frac{\mathrm{precision}\times \mathrm{recall}}{\mathrm{precision}+\mathrm{recall}},$$

where TP are the true positive edges, FP are the false positive edges and FN are the false negative edges.

The results are depicted in Table 1 and the presented values are annotated with a 95% confidence interval, over five trials. The tDBN+LL and tDBN+MDL denote, respectively, the tDBN learning algorithm with LL and MDL criteria. Similarly, the bcDBN+LL and bcDBN+MDL denote, respectively, the bcDBN learning algorithm with LL and MDL scoring functions.

Considering Network 1, the tDBN recovers a significantly lower number of edges, giving raise to lower recalls and similar precisions, when comparing with bcDBN for LL and MDL. The bcDBN+LL and bcDBN+MDL have similar performances. For N=2000, bcDBN+LL and bcDBN+MDL are able to recover in average 99% of the total number of edges.

For Networks 2 and 5, considering incomplete networks, the tDBN has again lower recalls and similar precisions than bcDBN. However, in this case, the bcDBN+MDL clearly outperforms bcDBN+LL for all number of instances *N* considered.

Moreover, in Network 5, taking a maximum intra-slice in-degree k=3, bcDBN only recovers 84% of the total number of edges, for N=2000. These results suggest that a considerable number of observations are necessary to fully reconstruct the complex BFS-consistent *k*-structures.

Curiously, the bcDBN+MDL algorithm has better results considering a complete tree-augmented initial structure (Network 4), with higher precision scores and similar recall, comparing with tDBN+MDL.

For both algorithms, in general, the LL gives raise to better results, when considering a complete network structure and a lower number of instances, whereas taking an incomplete network structure and a higher number of instances, the MDL outperforms LL. The complexity penalization term of MDL prevents the algorithms of choosing false positive edges and gives raise to higher precision scores. The LL selects more complex structures, such that each node has exactly p+k parents.

**Figure 6:**
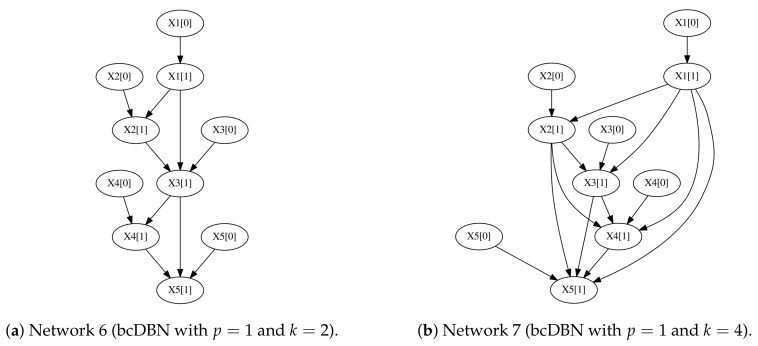
Two additional transition networks to test structure recovery in terms of number of observations *N*.

We stress that in all settings considered both algorithms improve their performance when increasing the number of observations *N*. In order to understand the number of instances *N* needed to fully recover the initial transition network, we designed two new experiments where five samples where generated from the first-order Markov transition networks depicted in Figure 6.

The number of observations needed for the bcDBN+MDL to recover the aforementioned networks are 1120.0±478.18 (Figure 6a) and 2900.0±1134.77 (Figure 6b), with a 95% confidence interval, where the five trials were done for each network. When increasing *k*, the number of necessary observations to totally recover the initial structure increases significantly.

When considering more complex BFS-consistent *k*-structures, the bcDBN algorithm achieved consistently significantly higher $F_{1}$ measures than tDBN. As expected, bcDBN+LL obtained better results for complete structures, whereas bcDBN+MDL achieved better results for incomplete structures.

## 7. Conclusions

The bcDBN learning algorithm has polynomial-time complexity with respect to the number of attributes and can be applied to stationary and non-stationary Markov processes. The proposed algorithm increases the search space exponentially, in the number of attributes, comparing with the state-of-the-art tDBN algorithm. When considering more complex structures, the bcDBN is a good alternative to the tDBN. Although a higher number of observations are necessary to fully recover the transition network structure, bcDBN is able to recover a significantly larger number of dependencies and surpasses, in all experiments, the tDBN algorithm in terms of $F_{1}$-measure.

A possible line of future research is to consider hidden variables and incorporate a structural Expectation-Maximization procedure in order to generalize hidden Markov models. Another possible path to follow is to consider mixtures of bcDBNs, both for classification and clustering.

## Figures and Tables

**Table 1:** Comparative structure recovery results for tDBN and bcDBN on simulated data. For each network, *n* is the number of network attributes, *p* is the maximum inter-slice in-degree, *k* is the maximum intra-slice in-degree, and *r* is the number of states of all attributes. On the left, *N* is the number of observations. Precision (Pre.), recall (Rec.) and $F_{1}$-measure ($F_{1}$) values are presented as percentages, running time is in seconds.

*N*	tDBN+LL				tDBN+MDL				bcDBN+LL				bcDBN+MDL			
	Pre.	Rec.	$F_{1}$	Time	Pre.	Rec.	$F_{1}$	Time	Pre.	Rec.	$F_{1}$	Time	Pre.	Rec.	$F_{1}$	Time
Network 1 (n=5,p=2,k=2,r=2)																
100	74±12	69±11	72±12	0	91±7	56±8	69±7	0	74±16	84±18	79±16	0	97±5	43±9	58±9	0
500	83±3	77±3	80±3	0	98±3	73±5	84±4	0	84±5	95±6	89±5	0	98±3	91±8	94±6	0
1000	81±5	76±5	79±5	0	98±3	79±2	87±3	0	85±4	96±5	90±4	0	97±5	96±7	97±6	0
2000	83±5	77±5	80±5	0	95±8	77±5	85±6	0	87±2	99±2	93±2	0	98±4	99±2	98±3	0
Network 2 (n=10,p=2,k=2,r=2)																
100	16±5	29±8	20±6	0	31±9	24±5	27±7	0	18±4	41±9	25±5	0	36±12	13±5	18±7	0
500	28±4	51±7	36±5	0	58±5	45±4	51±4	0	28±6	65±13	39±8	2	81±9	49±8	61±9	2
1000	33±3	60±6	43±4	0	61±5	46±4	52±5	0	32±4	75±8	45±5	4	66±7	55±9	60±8	4
2000	38±2	69±3	49±2	0	72±4	60±3	65±3	0	32±3	75±6	45±4	9	77±12	73±7	74±9	9
Network 3 (n=10,p=2,k=3,r=2)																
100	26±1	26±2	26±2	0	54±13	24±5	33±7	0	25±7	40±11	31±9	1	59±16	24±7	34±10	1
500	43±6	45±6	44±6	0	71±11	39±7	50±8	0	48±6	75±9	58±7	8	75±15	55±14	63±14	8
1000	43±6	44±6	44±6	0	68±7	41±6	51±6	0	47±6	74±9	58±7	18	75±10	61±7	67±8	18
2000	44±5	46±5	45±5	0	77±3	49±1	60±1	0	46±8	72±13	56±10	37	82±3	77±3	80±3	35
Network 4 (n=10,p=2,k=1,r=2)																
100	45±10	65±14	53±11	0	57±7	49±8	52±8	0	45±10	65±14	53±11	0	85±9	47±10	60±9	0
500	58±4	84±5	69±4	0	85±3	86±4	86±3	0	58±4	84±5	69±4	0	100±0	84±3	91±2	0
1000	63±1	92±2	75±2	0	88±2	91±3	90±3	0	63±1	92±2	75±2	0	100±0	88±2	94±1	0
2000	61±4	88±6	72±5	0	87±3	92±2	89±2	0	61±4	88±6	72±5	1	100±0	90±0	95±0	1
Network 5 (n=10,p=2,k=3,r=2)																
100	32±9	43±12	37±10	0	55±14	31±7	39±9	0	22±6	45±12	30±8	1	69±13	19±8	29±11	1
500	49±4	65±6	56±5	0	80±4	57±4	67±4	0	36±4	72±8	48±5	8	92±5	56±2	70±2	9
1000	50±6	66±7	57±6	0	83±6	64±5	72±5	0	40±4	79±9	53±6	16	90±8	71±9	79±8	17
2000	54±6	71±7	61±6	0	86±5	70±5	77±5	0	40±3	81±7	54±5	33	91±6	84±5	87±5	34

## References

[1] Pearl J. (2014). Probabilistic Reasoning in Intelligent Systems: Networks of Plausible Inference.

[2] Murphy K.P., Russell S. (2002). Dynamic Bayesian Networks: Representation, Inference and Learning.

[3] Yao X.Q., Zhu H., She Z.S. (2008). A dynamic Bayesian network approach to protein secondary structure prediction. BMC Bioinform..

[4] Zweig G., Russell S. (1998). Speech Recognition with Dynamic Bayesian Networks. Ph.D. Thesis.

[5] Van Gerven M.A., Taal B.G., Lucas P.J. (2008). Dynamic Bayesian networks as prognostic models for clinical patient management. J. Biomed. Inform..

[6] Friedman N., Geiger D., Goldszmidt M. (1997). Bayesian Network Classifiers. Mach. Learn..

[7] Grossman D., Domingos P. Learning Bayesian network classifiers by maximizing conditional likelihood. Proceedings of the Twenty-First International Conference on Machine Learning.

[8] Carvalho A.M., Roos T., Oliveira A.L., Myllymäki P. (2011). Discriminative Learning of Bayesian Networks via Factorized Conditional Log-Likelihood. J. Mach. Learn. Res..

[9] Carvalho A.M., Adão P., Mateus P. (2013). Efficient Approximation of the Conditional Relative Entropy with Applications to Discriminative Learning of Bayesian Network Classifiers. Entropy.

[10] Carvalho A.M., Adão P., Mateus P. (2014). Hybrid learning of Bayesian multinets for binary classification. Pattern Recognit..

[11] Dojer N. (2006). Learning Bayesian networks does not have to be NP-hard. International Symposium on Mathematical Foundations of Computer Science.

[12] Vinh N.X., Chetty M., Coppel R., Wangikar P.P. Polynomial time algorithm for learning globally optimal dynamic Bayesian network. Proceedings of the International Conference on Neural Information Processing.

[13] Monteiro J.L., Vinga S., Carvalho A.M. Polynomial-time algorithm for learning optimal tree-augmented dynamic Bayesian networks. Proceedings of the Conference on Uncertainty in Artificial Intelligence (UAI).

[14] Chow C., Liu C. (1968). Approximating discrete probability distributions with dependence trees. IEEE Trans. Inf. Theory.

[15] Dasgupta S. Learning Polytrees. Proceedings of the Fifteenth Conference on Uncertainty in Artificial Intelligence.

[16] Carvalho A.M., Oliveira A.L. Learning Bayesian networks consistent with the optimal branching. Proceedings of the Sixth International Conference on Machine Learning and Applications.

[17] Carvalho A.M., Oliveira A.L., Sagot M.F. Efficient learning of Bayesian network classifiers. Proceedings of the Australasian Joint Conference on Artificial Intelligence.

[18] Koller D., Friedman N. (2009). Probabilistic Graphical Models: Principles and Techniques.

[19] Rissanen J. (1985). Minimum Description Length Principle.

[20] Cooper G.F. (1990). The computational complexity of probabilistic inference using Bayesian belief networks. Artif. Intell..

[21] Chickering D.M. (1996). Learning Bayesian networks is NP-complete. Learning from Data.

[22] Dagum P., Luby M. (1993). Approximating probabilistic inference in Bayesian belief networks is NP-hard. Artif. Intell..

[23] Heckerman D., Geiger D., Chickering D.M. (1995). Learning Bayesian networks: The combination of knowledge and statistical data. Mach. Learn..

[24] Cooper G.F., Herskovits E. (1992). A Bayesian method for the induction of probabilistic networks from data. Mach. Learn..

[25] Friedman N., Murphy K., Russell S. Learning the structure of dynamic probabilistic networks. Proceedings of the Fourteenth Conference on UAI.

[26] Murphy K.P. (2001). The Bayes Net Toolbox for MATLAB. Comput. Sci. Stat..

[27] Edmonds J. (1968). Optimum branchings. Math. Decis. Sci..

